# Supplementary material for: Fall Prediction Based on Instrumented Measures of Gait and Turning in Daily Life in People with Multiple Sclerosis
Source: Sensors (Basel). 2022 Aug 9;22(16):5940. doi: 10.3390/s22165940 (PMC9415310; doi:10.3390/s22165940)
Supplement: Supplementary file 1 [file sensors-22-05940-s001.zip › sensors-1834541-supplementary.pdf]

Table S1. Mobility measures and their definitions grouped by domains of mobility. Each domain is color-coded.

Mobility Measures	Definition
QUALITY	
**Lower Body**	
Gait Speed (m/s)	The forward speed of the subject, measured as the forward distance traveled during the gait cycle divided by the stride duration.
Stride Length (m)	The forward distance traveled by the foot during a step.
Cadence (strides/min)	The number of strides per minute.
Stride Duration (s)	The duration from the time of initial contact to the time of the next initial contact of the same foot.
Double-Support (%)	The portion of the stride duration in which both feet are in contact with the ground, calculated as the sum of initial and terminal double-support as a percentage of the stride duration.
Swing (%)	The portion of the stride duration in which the foot is not in contact with the ground, calculated as the period from the time of toe-off until the time of initial contact as a percentage of the stride duration.
Elevation at Mid-Swing (cm)	The height of the foot sensor at the moment of maximum forward velocity of the foot, relative to the sensor height during stance.
Pitch at Toe-Off (°)	The angle of the foot relative to a level, horizontal surface at the time the foot leaves the ground.
Pitch at Initial Contact (°)	The angle of the foot relative to a level, horizontal surface at the time the foot begins contact with the ground.
**Upper Body**	
Transverse Range of Motion (rad)	The angular range torso movement at the lumbar level in the transverse plane.
Sagittal Range of Motion (rad)	The angular range of torso movement at the lumbar level in the sagittal plane.
Coronal Range of Motion (rad)	The angular range of torso movement at the lumbar level in the coronal plane.
**Turning**	
Turn Duration (s)	The time from the start of the turn until the end of the turn.
Turn Rate Average (°/s)	Average of the heading angle velocity of the lumbar torso during the turn period.
Turn Angle (°)	Total change in the heading angle of the turn calculated as the difference between the ending and starting heading angle.
Steps in Turn (#)	Total number of steps detected during the turn.
**Variability**	
All Mobility Measures Except Activity Domain	The coefficient of variation (CV) (standard deviation divided by the mean) across all the strides during a week of recording.
QAUNTITY	
**Activity**	
Strides per Hour (#)	The total number of stride pairs detected and validated during normal forward walking gait across all recordings divided by the total duration of all the recordings.
Turns per Hour (#)	The total number of detected turns across all recordings divided by the total duration of all the recordings.
Bouts per Hour (#)	The total number of bouts detected and validated during normal forward walking gait across all recordings divided by the total duration of all the recordings.

Table S2. Instrumented gait and turning measures collected during the daily home monitoring that were not significantly different between fallers and non-fallers.

	Faller/Non-Faller	N	Mean	SE	p
**CV for Gait Speed**	Non-Fallers	13	0.24	0.01	0.74
	Fallers	13	0.24	0.02	
**CV for Stride Length**	Non-Fallers	13	0.17	0.01	0.62
	Fallers	13	0.18	0.01	
**CV for Cadence**	Non-Fallers	13	0.12	0.01	0.72
	Fallers	13	0.12	0.01	
**CV for Stride Duration**	Non-Fallers	13	0.15	0.01	0.96
	Fallers	13	0.14	0.01	
**CV for Double Support**	Non-Fallers	13	0.19	0.02	0.53
	Fallers	13	0.20	0.02	
**CV for Swing**	Non-Fallers	13	0.06	0.01	0.07
	Fallers	13	0.09	0.01	
**CV for Elevation at Mid Swing**	Non-Fallers	13	0.73	0.06	0.31
	Fallers	13	0.64	0.06	
**CV for Pitch at Toe Off**	Non-Fallers	13	0.13	0.01	0.07
	Fallers	13	0.17	0.02	
**CV for Pitch at Initial Contact**	Non-Fallers	13	0.32	0.03	0.23
	Fallers	13	0.44	0.09	
**CV for Turn Duration**	Non-Fallers	12	0.36	0.01	0.60
	Fallers	13	0.37	0.01	
**CV for Turn Rate Average**	Non-Fallers	12	0.35	0.01	0.48
	Fallers	13	0.34	0.01	
**CV for Turn Angle**	Non-Fallers	12	0.49	0.01	0.47
	Fallers	13	0.48	0.01	
**CV for Steps in Turn**	Non-Fallers	12	0.59	0.03	0.86
	Fallers	13	0.58	0.02	
**CV for Transverse Range of Motion**	Non-Fallers	11	0.49	0.09	0.40
	Fallers	13	0.60	0.09	
**CV for Sagittal Range of Motion**	Non-Fallers	11	0.52	0.06	0.67
	Fallers	13	0.56	0.08	
**CV for Coronal Range of Motion**	Non-Fallers	11	0.35	0.05	0.66
	Fallers	13	0.38	0.04	
